# Acute Kidney Injury in Transcatheter Aortic Valve Replacement

**DOI:** 10.7759/cureus.15154

**Published:** 2021-05-21

**Authors:** Sakina Abbas, Ihtisham Qayum, Rabiya Wahid, FNU Salman, Henna Khan, Fatima Hassan, Anum Babar, Arslan Inayat

**Affiliations:** 1 Medicine, Dow University of Health Sciences, Karachi, PAK; 2 Internal Medicine, Khyber Teaching Hospital, Peshawar, PAK; 3 Medicine, Liaquat University of Medical and Health Sciences, Hyderabad, PAK; 4 Medicine, St. Vincent Medical Center, Toledo, USA; 5 Internal Medicine, Khyber Girls Medical College, Peshawar, PAK; 6 Medicine, Fatima Jinnah Medical University, Lahore, PAK; 7 Medicine, Khyber Girls Medical College, Peshawar, PAK; 8 Internal Medicine, University at Buffalo, Catholic Health System, Buffalo, USA

**Keywords:** tavr, tavi, aortic stenosis, transcatheter aortic valve replacement, transcatheter aortic valve implantation

## Abstract

Introduction

Transcatheter aortic valve replacement (TAVR) has been established as a standard of care for patients with severe aortic stenosis. We aim to study the predictors of acute kidney injury (AKI) after TAVR from a contemporary analysis using the National Inpatient Sample (NIS) database.

Methods

We performed a national analysis using the NIS database to evaluate predictors of acute kidney injury (AKI) after TAVR. Our study period was from 2015 to 2018, and we identified TAVR patients in all procedure fields. Patients aged less than 18 years were excluded from the study.

Results

We report data of 173,760 TAVR patients, of which 20,045 (11.5%) had AKI and 153,715 (88.4%) did not. There were three principal findings of our study. First, mortality was higher in patients with AKI compared to patients who did not have AKI (8% vs. 0.8%; p<0.01). Second, patients with chronic kidney disease, weight loss, liver disease, congestive heart failure, cerebrovascular disease, chronic obstructive pulmonary disease, metastatic cancer, and peripheral vascular disease had higher adjusted odds of AKI. Third, length of stay and cost of stay were significantly higher in patients who had AKI during the index admission.

Conclusion

Patients with AKI had higher in-hospital mortality. We also report that at baseline, chronic kidney disease, weight loss, liver disease, congestive heart failure, cerebrovascular disease, chronic obstructive pulmonary disease, metastatic cancer, and peripheral vascular disease were important predictors of AKI in patients after TAVR. Length of stay and cost of stay were higher with AKI, which result in higher burden on the health care system due to increased resource utilization.

## Introduction

Transcatheter aortic valve replacement has revolutionized the treatment of patients with severe aortic stenosis who are considered high risk for surgical aortic valve replacement [1-4]. The PARTNER (Placement of AoRTic TraNscathetER Valves) trials have led the way for broadening the indication of TAVR to low-risk surgical patients [3]. TAVR has led to a constant improvement in clinical outcomes with aortic stenosis [5]. In patients with aortic valve disease, TAVR, in the last 15 years has progressed from a last resort procedure in patients who were at high perioperative risk for major mortality and morbidity from surgical valve replacement to a viable and alternate option to surgery [6]. Although TAVR has shown great results, acute kidney injury (AKI) remains one of the major complications and is associated with mortality, increased adverse events, and resource use [7,8].

Rapid loss of kidney function occurring within hours or days and resulting in impaired electrolyte hemostasis, dysregulation of volume, and accumulation of waste product is defined as AKI [9]. AKI is a frequent complication seen in TAVR that remains associated with a dismal prognosis and was identified as one of the most common complications in the landmark PARTNER trials [2,3,10-12]. According to one estimate, AKI is seen in up to 30% of patients undergoing TAVR [13]. We know from prior literature that AKI in surgical aortic valve replacement was associated with up to a four-fold higher risk of mortality. During the procedure, there are multiple predisposing factors such as contrast use and hypotension episodes that predispose to acute renal injury [14]. As TAVR is mostly performed in elderly and frail patients, CKD has a wide prevalence and further predisposes patients to acute-on-chronic kidney injury [12,14-18].

Previous studies have looked at AKI in TAVR; however, studies have remained limited to case series and retrospective studies with a small sample size. Predictors of AKI after TAVR from a large sample size of patients and real-world experience of TAVR remain scarce. Hence, the aim of this study was to identify important predictors of AKI, mortality rate, and resource utilization after TAVR from a U.S. national database.

## Materials and methods

We used the U.S. National Inpatient Sample (NIS) database to identify cases of TAVR performed from 2015 to 2018. The NIS data are available to the public and anonymized; hence, Institutional Review Board approval was not necessary for this study.

To perform a national analysis, we used the International Classification of Diseases (ICD)-9 and ICD-10 codes (3505, 3506, and O2RF3) to identify hospitalizations for TAVR. We queried all diagnosis fields to select TAVR patients. Similarly, ICD-9 and ICD-10 diagnosis codes were used to define and identify all baseline co-morbidities. We excluded patients who were less than 18 years of age.

We used weighted data based on discharge weights provided by the NIS. For categorical variables, we used the chi-square test. For continuous variables, testing of non-normality was used. Since continuous variables are not normally distributed, Mann-Whitney U test was used. We also developed a binary logistic model using entry method including demographic factors such as age, sex, race, median income, hospital location, baseline co-morbidities, obesity, weight loss, metastatic cancer, lymphoma, solid organ tumor, alcohol use, coagulopathy, hypothyroidism, chronic obstructive pulmonary disease (COPD), cerebrovascular disease (CVA), congestive heart failure (CHF), coronary artery disease (CAD), diabetes mellitus, hypertension, liver disease, chronic kidney disease (CKD), and peripheral vascular disease (PVD). In compliance with the Healthcare Cost and Utilization Project guidelines, we did not report observations with less than 11 cases. Comparisons were two-sided, and p < 0.05 was considered statistically significant. All analyses were performed utilizing SPSS Version 27 (IBM Corp., Armonk, NY) and R Version 3.5 (R Foundation for Statistical Computing, Vienna, Austria).

## Results

**Figure 1:**
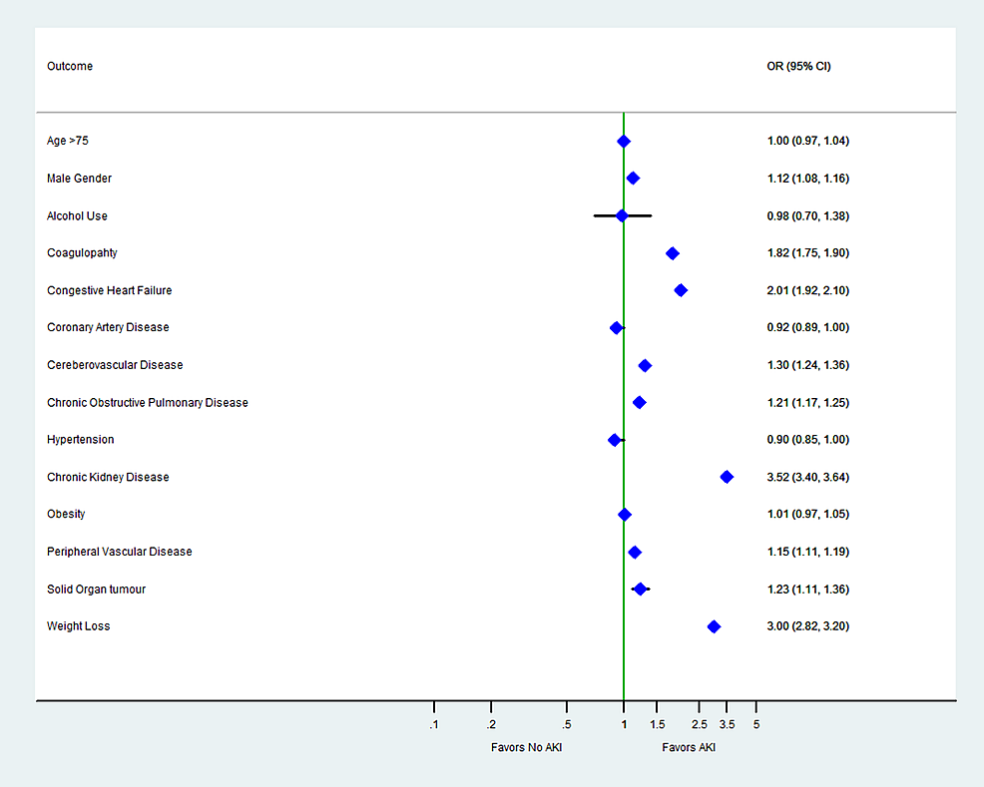
Adjusted odds of predictors of in-hospital AKI in patients with TAVR AKI, acute kidney injury; TAVR, transcatheter aortic valve replacement

A total of 173,760 weighted hospitalizations for TAVR were included in the analysis. Of the patient undergoing the procedure, 20,045 (11.5%) had AKI and 153,715 (88.4%) did not. The detailed baseline characteristics are summarized in Table 1. At baseline, patients with CKD (OR: 3.52; 95% CI: 3.40-3.64), weight loss (OR: 3.01; 95% CI: 2.82-3.20), liver disease (OR: 2.29; 95% CI: 2.13-2.46), CHF (OR: 2.01; 95% CI: 1.92-2.10), CVA (OR: 1.30; 95% CI: 1.24-1.36), COPD (OR: 1.21; 95% CI: 1.17-1.25), metastatic cancer (OR: 1.16; 95% Cl: 0.95-1.41), and PVD (OR: 1.15; 95% CI: 1.11-1.19) had higher adjusted odds of AKI (Table 1, Figure 1). Mortality rate was higher with AKI (8% vs 0.8%; p<0.01). Patients who had AKI had a higher median cost of stay (US$63,110 vs. US$44,853; p<0.01) and length of stay (9 vs. 2 days; p<0.01) (Table 2).

**Table 1:** Baseline characteristics and predictors of AKI in patients after TAVR AKI, acute kidney injury; IQR, interquartile range; TAVR, transcatheter aortic valve replacement

Variable	No AKI (153,715)	With AKI (20,045)	No AKI vs. AKI (multivariate analysis), OR (95% CI)
Age, median (IQR)	82 (75-86)	82 (74-87)	1.00 (0.97-1.04) (reference age < 75 years)
Male gender	81590 (53.1%)	11585 (57.8%)	1.12 (1.08-1.16) (reference to female)
Caucasian	128800 (87.4%)	15875 (83.4%)	Reference
African Americans	6060 (4.1%)	1005 (5.3%)	0.74 (0.67-0.81)
Hispanics	6855 (4.6%)	1285 (6.7%)	0.71 (0.63-0.80)
Chronic kidney disease	33645 (21.9%)	10575 (52.8%)	3.52 (3.40-3.64)
Weight loss	3870 (2.5%)	1865 (9.3%)	3.01 (2.82-3.20)
Liver disease	4060 (2.6%)	1405 (7.0%)	2.29 (2.13-2.46)
Congestive heart failure	110385 (71.8%)	17205 (85.8%)	2.01 (1.92-2.10)
Coagulopathy	17270 (11.2%)	4315 (21.5%)	1.82 (1.75-1.90)
Cerebrovascular disease	17320 (11.3%)	2930 (14.6%)	1.30 (1.24-1.36)
Solid organ tumor	3655 (2.4%)	605 (3.0%)	1.23 (1.11-1.36)
Chronic obstructive pulmonary disease	46145 (30.0%)	7095 (35.4%)	1.21 (1.17-1.25)
Metastatic cancer	995 (0.6%)	150 (0.7%)	1.16 (0.95-1.41)
Peripheral vascular disease	33015 (21.5%)	5130 (25.6%)	1.15 (1.11-1.19)
Obesity	25870 (16.8%)	3335 (16.6%)	1.01 (0.97-1.05)
Lymphoma	1060 (0.7%)	125 (0.6%)	0.99 (0.81-1.20)
Alcohol use	240 (0.2%)	45 (0.2%)	0.98 (0.70-1.39)
Hypothyroidism	31350 (20.4%)	4050 (20.2%)	0.98 (0.94-1.02)
Coronary artery disease	106475 (69.3%)	13950 (69.6%)	0.92 (0.89-1.00)
Hypertension	136605 (88.9%)	18015 (89.9%)	0.90 (0.85-1.0)
Diabetes mellitus	25140 (16.4%)	1990 (9.9%)	0.76 (0.72-0.80)
Income			
0-25th percentile	32110 (21.2%)	4485 (22.7%)	Reference
25th-50th percentile	38530 (25.4%)	5210 (26.4%)	1.08 (1.03-1.13)
50th-75th percentile	40805 (26.9%)	4955 (25.1%)	1.05 (1.00-1.10)
75th-100th percentile	39990 (26.4%)	5075 (25.7%)	0.93 (0.89-0.97)
Urban	1380 (0.9%)	110 (0.5%)	Reference
Urban non-teaching	14550 (9.5%)	1625 (8.1%)	0.56 (0.46-0.69)
Urban teaching	137785 (89.6%)	18310 (91.3%)	0.85 (0.80-0.90)

 

**Table 2:** In-hospital outcomes of patients with AKI in TAVR AKI, acute kidney injury; IQR, interquartile range; TAVR, transcatheter aortic valve replacement

Outcome	No AKI	AKI	p-Value
Died during hospitalization, n (%)	1285 (0.8%)	1605 (8%)	<0.01
Length of stay, median (IQR)	2 (2-4)	9 (5-15)	<0.01
Cost of stay (in US dollars), median (IQR)	44853 (35218-56473)	63110 (47235-85623)	<0.01

## Discussion

The principal findings of our study are summarized as follows: (1) AKI occurs in about 11.5% of patients undergoing TAVR, (2) mortality is higher in patients with AKI compared to patients who do not have AKI (8% vs. 0.8%; p<0.01), (3) factors at baseline such as male sex, CVA, CHF, COPD, liver disease, CKD, metastatic cancer, weight loss, and PVD are important predictors of AKI in patients undergoing TAVR, and (4) patients who developed AKI after TAVR have a higher length of stay and cost of stay.

The reported incidence of post-TAVR AKI is 22.1% ± 11.2% based on the current Valve Academic Research Consortium (VARC)-2. Both persistent and transient AKI have been independently associated with higher mortality rates. Hence, our study aims to identify baseline characteristics that predispose a patient to AKI so that we can institute an approach of pre-TAVR intravenous fluid hydration and try to minimize contrast exposure in these patients [9]. In contrast to the previous literature, in our national analysis, we have reported an AKI rate of 13%. However, it is important to note that we used ICD codes to identify AKI, whereas prior literature has reported AKI in terms of VARC-2 criteria [12]. However, our study results were congruent with the findings of Bagur et al., who reported an AKI rate of 11.7%, which is very close to our estimate of 13%. In prior literature, mortality rate in TAVR was approximated to be 28%, which was significantly higher compared to our study findings (8%) [14]. One of the possible explanations is that our study was more recent and has looked at TAVR experience in recent times, which have undergone significant technological advancements and increased operator experience. Similarly, Aregger et al. reported data on a series of 54 patients and reported an AKI frequency of 28% [19]. In contrast, our study reported more new findings from a large sample size of 173,760 patients, which would be a better indicator of AKI complication rates in recent times.

According to one estimate, patients who developed AKI had a four-fold increase in mortality. Kidney disease is a well-known predictor of worse outcomes in patients undergoing TAVR [12,20,21]. Our study findings are consistent with prior literature, and we report that patients with CKD have a 3.5 times higher risk of developing AKI. Similarly, co-morbidities such as COPD have been reported in prior literature to cause AKI [22]. Our study reinforces these findings and reports a 1.12-fold higher risk of AKI with COPD. Similarly, our national analysis identifies CVA, liver disease, and PVD as significant predictors of this adverse event. Previous studies have reported hypertension to be an important predictor of AKI due to loss of kidney autoregulation. However, in our analysis, hypertension was not found to be significantly associated with an increased incidence of AKI [23].

Our study findings must be interpreted in light of the following limitations. This was a retrospective study hence association should not be misinterpreted as causation. Important data including medication use and laboratory data were not available in the NIS. Surgical risk scores such as EuroSCORE (European System for Cardiac Operative Risk Evaluation) and STS (Society of Thoracic Surgeons) score cannot be calculated using this database. We were only able to look at in-hospital data, and longer follow-up data were not available. NIS is a billing dataset that relies on ICD codes; hence, coding errors are a possibility. CKD in our study was defined as CKD-1 to CKD-IV based on ICD-9 and ICD-10 diagnosis codes. We used ICD codes whereas non-database studies used standardized definitions. However, we used well-validated ICD codes that reduce the chance of coding errors. We are not able to quantify the degree of AKI from the NIS database given its inherent limitations; in contrast, previous studies used VARC-2 criteria for AKI definition and were able to quantify AKI.

## Conclusions

In conclusion, our study reported a contemporary analysis of 173,760 TAVR patients. The rate of AKI post-TAVR was 11.5%. We further reported that at baseline CKD, weight loss, liver disease, CHF, CVA, COPD, metastatic cancer, and PVD were important predictors of AKI in patients undergoing TAVR. AKI in TAVR is associated with increased burden on the healthcare system in the form of increased length of hospital stay and cost of hospitalization. It is of utmost importance to identify patients who are at high risk of developing complications as TAVR indication expands to a larger population. These data can also help clinicians in decision-making to optimize patients prior to the procedure and decrease the risk of AKI post-TAVR.
